# Ultrahigh Resolution Thickness Measurement Technique Based on a Hollow Core Optical Fiber Structure

**DOI:** 10.3390/s20072035

**Published:** 2020-04-04

**Authors:** Zheyu Wu, Bin Liu, Jiangfeng Zhu, Juan Liu, Shengpeng Wan, Tao Wu, Jinghua Sun

**Affiliations:** 1 School of Physics and Optoelectronic Engineering, Xidian University, Xi’an 710071, China; zheyu.wu1999@gmail.com; 2 National Engineering Laboratory for Destructive Testing and Optoelectronic Sensing Technology and Application, Nanchang Hang Kong University, Nanchang 330063, China; liubin@nchu.edu.cn (B.L.); 18042@nchu.edu.cn (J.L.); spwan@nchu.edu.cn (S.W.); wutccnu2001@aliyun.com (T.W.); 3 Sch Elect Engn & Intelligentizat, Dongguan University of Technology, Dongguan 523808, China; sunjh@dgut.edu.cn

**Keywords:** optical fiber sensing, hollow core optical fiber, graphene oxide (GO), thickness measurement

## Abstract

An ultrahigh resolution thickness measurement sensor was proposed based on a single mode–hollow core–single mode (SMF–HCF–SMF) fiber structure by coating a thin layer of material on the HCF surface. Theoretical analysis shows that the SMF–HCF–SMF fiber structure can measure coating thickness down to sub-nanometers. An experimental study was carried out by coating a thin layer of graphene oxide (GO) on the HCF surface of the fabricated SMF–HCF–SMF fiber structure. The experimental results show that the fiber sensor structure can detect a thin layer with a thickness down to 0.21 nanometers, which agrees well with the simulation results. The proposed sensing technology has the advantages of simple configuration, ease of fabrication, low cost, high resolution, and good repeatability, which offer great potential for practical thickness measurement applications.

## 1. Introduction

Dielectric coating of optical fibers has been widely applied to various sensing applications and has been shown to improve the performance of fibers. Optical fibers coated with non-uniform metal coatings of gold, silver, copper or palladium exhibit better sensitivity to various forms of perturbations [1]. In particular, palladium (Pd) coated optical fiber sensors demonstrate an ultrahigh sensitivity for detecting hydrogen [2]. Polyimide coated optical fibers can effectively respond to humidity changes [3]. Zinc oxide (ZnO) coated long-period fiber gratings (LPFG) have improved refractive index (RI) sensing properties [4]. In certain cases, the accuracy of the coating thickness is very important for the qualitative study of the physical properties. Various methods for the accurate measurement of the coating thickness have been widely studied. A method has been proposed for measuring thin film thickness using total internal reflection fluorescence microscopy with the use of evanescent wave illumination, which can overcome the drawbacks of the optical methods that are insufficient for measuring the thickness of a thin film with curvature [5]. A scanning angle (SA) Raman spectroscopy method enables the non-destructive measurement of the polymer thickness [6,7]. High frequency ultrasonic transducers can be used to characterize the variation of thickness in polymer thin films (<1 µm) [8]. Reflection high-energy electron diffraction, piezoelectricity, interferometry, and gravimetric methods can be used to measure the thickness of films in the range of 1 nm‒1 µm during film deposition or on a finished product [9]. Scanning electron microscopy (SEM), which has a good resolution and simple operation, is most commonly used to detect the thickness of a coating material. However, SEM is expensive, and measurement is usually limited by the sample conductivity (for dielectrics, a thin layer metal coating is required) and requires high vacuum conditions [10]. Fourier transform-based structured-illumination microscopy (FTSIM) and modulation-based structured-illumination microscopy (MSIM) can also detect the surface topography and thickness distribution [11,12]. Fourier-domain optical coherence tomography (FDOCT) is an alternative method [13], however, this method is again costly and relatively complicated, and can only achieve micrometer-scale detection, which has a relatively low resolution. 

Hollow-core fiber (HCF) has attracted a lot of research interest in the field of optical fiber sensors. For instance, a simple structure is demonstrated for the refractive index sensing by splicing a segment of capillary with two segments of single mode fibers (SMFs) [14]. An air-gap microcavity is incorporated in the HCF to form a Fabry–Pérot interferometer (FPI) for sensing applications [15,16,17]. In FPI-based sensors, the size of the microcavity needs to be very accurately controlled to the order of microns, as subtle variations in the length of the cavity will result in significant changes in the actual spectrum. The resolution and detection limits for whispering gallery mode-based refractometric sensor devices can be quickly estimated by a simple numerical relationship [18]. Previous reports show that HCF-based interferometer structures have been used for temperature [19], vibration [20], and humidity sensing [21], demonstrating its wide application prospects.

In this paper, we propose and investigate a new way to use a single HCF-based multiple beam interferometer for thin-layer thickness measurement, in which a short section of HCF is fusion spliced between two SMFs. The proposed method can achieve an ultrahigh resolution with sub-nanometer thickness detection. The demonstrated sensor has the advantages of being low cost, having good repeatability, and an ease of fabrication.

## 2. Theoretical Analysis

**Figure 1:**
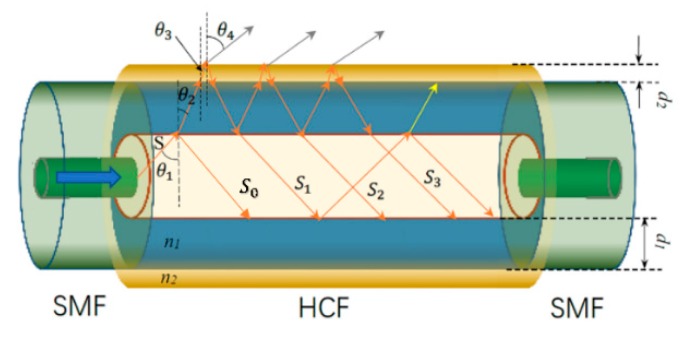
A schematic diagram of the coated single mode–hollow core–single mode (SMF–HCF–SMF) fiber structure.

A schematic diagram of the proposed HCF sensor for thickness measurement is shown in Figure 1. The sensor structure is an SMF–HCF–SMF fiber structure, and the thin layer that is to be measured is coated on the HCF section. When light is transmitted from the input SMF to the HCF, because the diameter of the SMF (around 8.2 µm) is smaller than the air core (around 30 µm) of the HCF and the RI of the silica cladding is larger than that of the air core, no guided mode is transmitted within the HCF. As illustrated in Figure 1, an obvious anti-resonant reflecting guidance mechanism is triggered in the HCFs [19,22,23], which relies on the principle of thin-film interference and is created by forming a Fabry–Pérot in the transverse direction, with cladding layers that function as Fabry–Pérot etalons [24,25]. The light that reaches the silica cladding of the HCF propagates within the cladding. The coated thin-layer material is equivalent to thickening the outer diameter of the HCF. The incident light from the SMF is reflected at both the interface between the inner air/cladding and the outer air/coated layer, resulting in multiple beam interferences within the HCF.

Provided that the input light (S) has an amplitude (A), the silica cladding thickness is $d_{1}$ and the RI is $n_{1}$, the coating thickness is $d_{2}$, and the RI is $n_{2}$. When the light is injected into the cladding, there is an incident angle of $\theta_{1}$ and a refractive angle of $\theta_{2}$, and when it is injected into the coating, there is an incident angle of $\theta_{2}$ and a refractive angle of $\theta_{3}$. Then, the phase difference between the two adjacent reflected light rays inside the air core of the HCF ($S_{0}$, $S_{1}$, $S_{2}$, $S_{3}$, etc.) is $\delta =\frac{4\pi }{\lambda }n_{1}d_{1}\cos\theta_{2}+\frac{4\pi }{\lambda }n_{2}d_{2}\cos\theta_{3}$. The complex amplitude of the reflected light can be expressed as $S_{0}=r_{1}A$ and $S_{p}=t_{1}t_{4}t_{2}^{p}t_{3}^{p}r_{2}^{p-1}r_{3}^{p}Ae^{ip\delta }\;\left(p\geq 1\right)$ [18]. $r_{1}$, $r_{2}$, and $r_{3}$ are the coefficients of reflection at the interfaces between the inner air/cladding, cladding/inner air, and coating/outer air, respectively, which can be calculated by the Fresnel equations, as follows:

(1) $$\mathrm{TE}\;\mathrm{mode}\;r_{1}=\frac{\cos\theta_{1}-n_{1}\cos\theta_{2}}{\cos\theta_{1}+n_{1}\cos\theta_{2}},\;\;r_{2}=-r_{1},\;\;r_{3}=\frac{n_{2}\cos\theta_{3}-\cos\theta_{4}}{n_{2}\cos\theta_{3}+\cos\theta_{4}}$$

(2) $$\mathrm{TM}\;\mathrm{mode}\;r_{1}=\frac{n_{1}\cos\theta_{1}-\cos\theta_{2}}{n_{1}\cos\theta_{1}+\cos\theta_{2}},\;\;r_{2}=-r_{1},\;\;r_{3}=\frac{\cos\theta_{3}-n_{2}\cos\theta_{4}}{\cos\theta_{3}+n_{2}\cos\theta_{4}}$$

where $\theta_{4}$ is the refractive angle between the coating/outer air, and $t_{1}$, $t_{2}$, $t_{3}$, and $t_{4}$ are the coefficients of refraction at the interface between the inner air/cladding, cladding/coating, coating/cladding, and cladding/inner air, respectively. Their relationship is as follows:

(3) $$t_{1}=t_{4},\;\;\;\;\begin{array}{cc} & t_{2}=t_{3} \end{array},\;\;\;\;\begin{array}{cc} & t_{1}^{2} \end{array}+r_{1}^{2}=1$$

If there are $p=L/2\left(d_{1}\tan\theta_{2}+d_{2}\tan\theta_{3}\right)$ reflected light rays, where *L* is the length of the coated HCF fiber, then the amplitude of reflected light after simplification can be expressed as follows:

(4) $$A_{r}=r_{1}A+t_{1}^{2}t_{2}^{2}r_{3}Ae^{i\delta }+t_{1}^{2}t_{2}^{4}r_{2}r_{3}^{2}Ae^{i2\delta }+\cdots +t_{1}^{2}t_{2}^{2p}r_{2}^{p-1}r_{3}^{p}Ae^{ip\delta }+\cdots ,$$

where the intensity of light reflected is thus $I_{r}={\left|A_{r}\right|}^{2}$.

**Figure 2:**
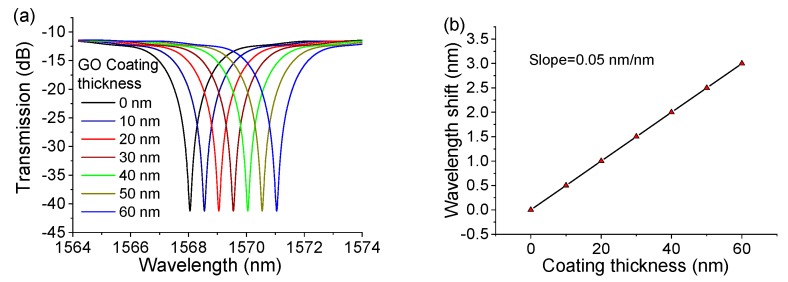
Simulated (**a**) spectral responses and (**b**) wavelength shift of the SMS–HCF–SMS fiber structure at different coating thicknesses.

The coating thickness, $d_{2}$, is inversely proportional to λ. Therefore, as the coating thickness increases, the anti-resonant reflecting guidance wavelength must be redshifted to keep the phase difference, δ, unchanged. Figure 2a shows the simulated spectral response for the different coating thicknesses of the structure. In the simulation, the diameters of the air core and silica cladding of the HCF are 30 and 126 μm, respectively; the RIs of the air core and cladding are 1.0 and 1.451, respectively; and the RI of the graphene oxide (GO) coating film is 1.9 [26]. As the coating thickness increases, the spectral response shifts to a longer wavelength linearly, with a change rate of 0.05 nm, as shown in Figure 2b. Assuming the optical spectrum analyzer (OSA) has a resolution of 0.01 nm, the theoretical limit of measurement for the GO coating layer is 0.2 nm. The simulated results indicate that the SMF–HCF–SMF fiber structure can measure thickness with ultrahigh resolution in the order of sub-nanometers. 

## 3. Experimental Results and Discussion

**Figure 3:**
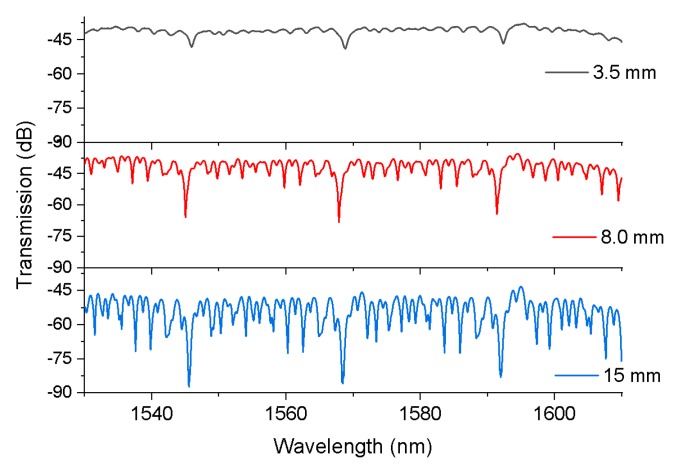
Measured transmission spectra of the SMF–HCF–SMF fiber structure with different lengths of HCF (3.5, 8.0, and 15 mm).

Experimental verification was carried out by fusion splicing a short section HCF (cap030/150/24t, fiber guide) between two traditional SMFs (G652D). In our experiment, the model of splicer machine employed was a Fujikura 80C. The manual splice mode was used to combine the HCF with the SMFs, and the discharge power, discharge time, and overlap length were standard +8 bit, 100 ms, and 9 μm, respectively. The HCF used in our experiments had core (air) and silica cladding diameters of 30 and 126 μm, respectively. The measured transmission spectral responses of the SMF–HCF–SMF fiber structure with HCF lengths of 3.5, 8, and 15 mm are shown in Figure 3. Figure 3 also shows that the wavelength of the anti-resonant dip was independent of the length of the HCF, but the extinction ratio of the anti-resonant dip increased with the length of the HCF. The optimal sample length of the HCF was chosen as 8.0 mm in the following experiments, for considering a better Q factor value of 1.2 × 10^4^. 

**Figure 4:**
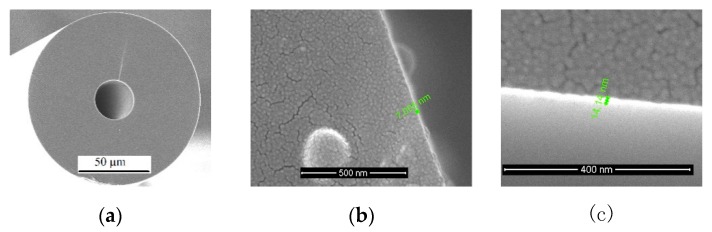

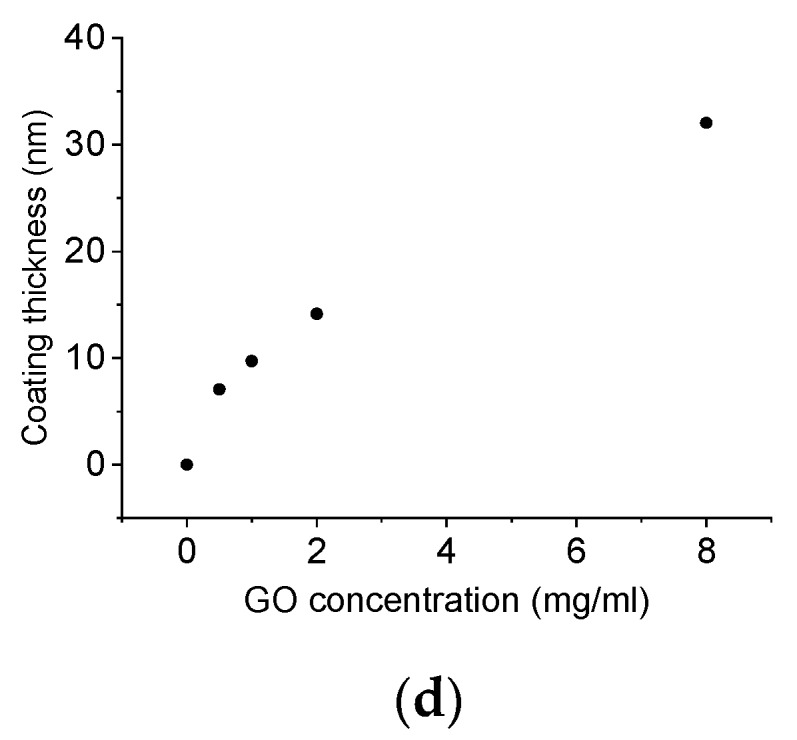
SEM image of the graphene oxide (GO) coated HCF (**a**) cross section, and the coating thickness of the GO layer with GO concentrations of (**b**) 0.5 mg/mL and (**c**) 2 mg/mL, and (**d**) the relationship between the coating thickness and GO concentration.

A typical nano-coating layer of thin graphene oxide (GO) was coated on the surface of the HCF, using the dip coating method of putting the HCF sensor into the GO solution and pulling it out with uniform speed (2 mm/s), and then drying it in air at room temperature to form a GO thin layer on the HCF surface for thickness measurement. The calibration of the coating thickness vs. the GO concentration was carried out using SEM. Figure 4a–c shows an example of the SEM image of the HCF cross section and GO-coated HCF with GO concentrations of 0.5 and 2 mg/mL. It can be seen from Figure 4b,c that the coating thicknesses were 7.005 nm and 14.14 nm for GO concentration of 0.5 and 2 mg/mL, respectively. In our coating thickness calibration experiment, four different coating thicknesses of 0.5, 1.0, 2.0, and 8.0 mg/mL were measured using SEM, and the relationship between the coating thickness and GO concentration is plotted in Figure 4d. As the GO concentration increased from 0.5 to 1, 2, and 8.0 mg/mL, the coating thickness increased monotonically from 7.005, 9.71, 14.14, and 32.04 nm, respectively, although the increase was not linear.

**Figure 5:**
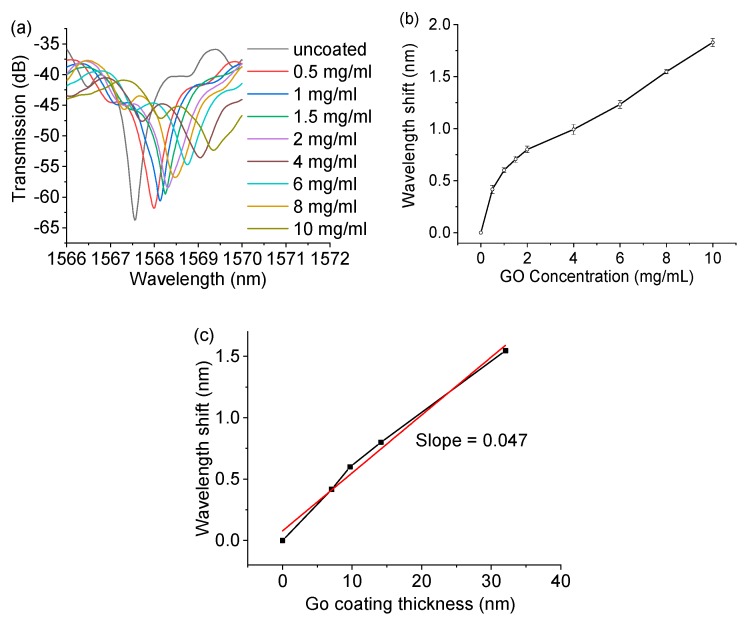
Measured (**a**) transmission spectra of the SMF–HCF–SMF fiber structure coated with different concentrations of GO, (**b**) wavelength shift vs. GO concentration, and (**c**) wavelength shift vs. GO coating thickness.

The influence of the concentration of the GO solution on the spectral response of the fiber sensor was carried out by dip coating the SMF–HCF–SMF fiber structure (HCF length of 8 mm) with eight different GO concentrations (0.5, 1, 1.5, 2, 4, 6, 8, and 10 mg/mL). The measured transmission spectral responses of the SMF–HCF–SMF fiber structure with different concentrations of GO are shown in Figure 5a. The wavelength of the anti-resonant dip had a monotonical redshift relative to the increase in concentration of GO. It is noted that as the GO concentration increased, the strength of the transmission dip decreased. This is possibly due to the fact that a higher concentration of GO solution will introduce a thicker coating on the HCF (resulting in a redshift of spectra), but the larger surface roughness, thickness, non-uniformity, and light scattering will result in a larger power difference between the light reflected by the coating/air interface and the inner air/cladding interface, thus reducing the strength of the dip wavelength [27].

Repeatability tests were carried out by coating the same concentration of GO on the same fiber sample five times—before each coating, the fiber was immersed into pure water, and the coated GO was washed out by the pure water to make sure it recovered to the original spectrum in air. The wavelength shifts of the anti-resonant dip are summarized in Figure 5b. The results in each GO concentration show that minor wavelength variations (error bar) were observed with the same concentration of GO, showing a very good repeatability of the sensor. Figure 5b also shows that as the GO concentration increased, the wavelength shifted to a longer wavelength monotonically, but there was no linear change. By applying the calibrated coating thickness vs. GO concentration in Figure 4d to Figure 5b, the relationship between the wavelength shifts vs. coating thickness is illustrated in Figure 5c. It shows that as the coating thickness increased, the wavelength shifted to a longer wavelength linearly, with a sensitivity of 0.047 nm. Assuming the OSA had a resolution of 0.01 nm, the calculated thickness resolution of the HCF sensor (based on the experimental results) could be estimated to be as low as 0.21 nm, which verified the simulated resolution of 0.2 nm in Figure 2.

**Figure 6:**
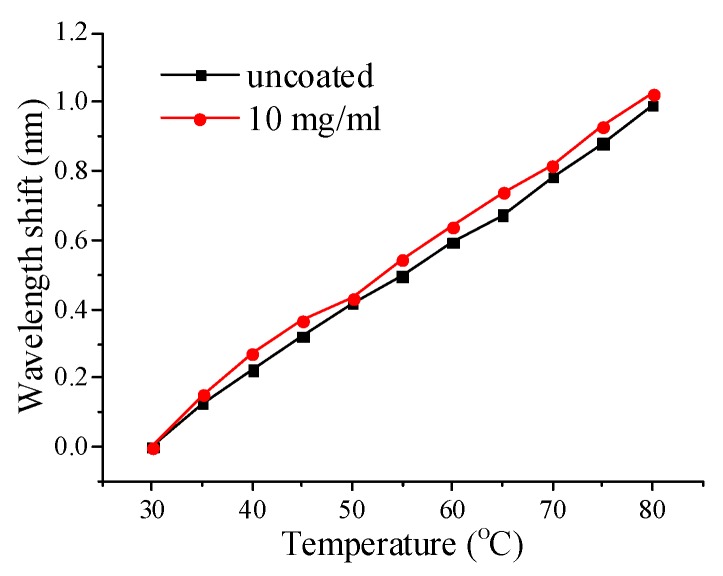
Wavelength shift vs. temperature for the SMF–HCF–SMF fiber structure with and without a coating of GO.

The influence of temperature was also investigated, and the result is shown in Figure 6. The sensors both uncoated and coated with a GO concentration of 10 mg/mL were chosen for the purpose of comparison. The sensors were characterized in terms of the anti-resonant wavelength shift as a function of temperature, over a temperature range from 30 to 80 °C. The temperature sensitivities for the coated and uncoated GO were 19.64 pm/°C and 19.2 pm/°C, respectively. The temperature response of the SMF–HCF–SMF fiber structure was almost unaffected by the GO coating. The reason is possibly because the coating thickness of GO is very thin, therefore the temperature influence is very limited.

## 4. Conclusion

In conclusion, an ultrahigh fiber sensor for measuring the thickness of nanometercoatings was proposed, based on an SMF–HCF–SMF structure. Both theoretical and experimental studies have demonstrated that the novel technique can measure very thin GO layer coatings, down to a sub-nanometer, with good repeatability. The temperature sensitivity of the sensor structure has also been studied experimentally, and the results show that the coating of GO has very limited influence on the sensitivity of the fiber structure. The proposed technique has other potential applications, such as measuring the viscosity and concentration of a material. For instance, it can measure the viscosity of a liquid by coating a thin layer of the material on the HCF surface, particularly for very low viscosity polymer materials. The sensor has the unique advantages of having ultrahigh resolution, simple fabrication, and good repeatability, as well as being low cost.

It is noted that although the analysis above is based on a dielectric material, the sensor can also be used for metal measurement, provided calibration has been taken for each type of metal. However, as shown in Figure 3 in the literature [27], because of the high transmission loss of metals, the extinction ratio reduces significantly as the metal coating thickness increases, resulting in a very narrow thickness measurement range (around 10 nm).
